# Extracurricular pursuits

**DOI:** 10.1192/bjb.2021.111

**Published:** 2022-06

**Authors:** Jonathan Williams

**Affiliations:** Consultant Child and Adolescent Psychiatrist, NHS North Central London, UK

**Keywords:** Psychodynamic, psychotherapy, dementia, CPD, extracurricular

## Abstract

Dr Dignan’s poetry, her care, and her enthusiasm should be lauded. There are also many other non-medical pursuits that may make us better doctors. But it is difficult to know which of these are effective or practicable.

In medical school we used to debate whether we would prefer to be a kind doctor or a clever one. Of course, most of us wanted to be both. But when we were ill ourselves, if we had to choose we were sure we would want a clever doctor to deal with serious conditions and a kind one for the minor things.

There is a related rift in mental health services between psychodynamic work and evidence-based medicine. Certainly, most patients are given one or the other. Corinne Dignan in her article^1^ focuses on something more on the psychodynamic side, that we are in danger of forgetting: the feelings and the shared experiences that she neatly calls the person behind the diagnostic labels. In this commentary I focus on this specific suggestion, rather than the general topic of medical humanities.

To clarify, she is not pushing us to become psychotherapists. She sees a beauty in each person, the special moments in each life and the treasured memories that gradually disappear in dementia. She experiences shared memories as special objects that will die unless a person keeps them alive. Indeed, as long as one person keeps them alive, part of the departed person still exists. In some senses they do; the memories exist, and we may sometimes feel that the person does too. This is a point that I've not seen so well treated elsewhere. Each experience, or at least an echo of it, continues to exist for as long as someone thinks about it (akin to the tree in the quad^2^). Dignan's article serves to warn us not to forget John Eccles's World 2 (subjective knowledge) as opposed to Worlds 1 and 3 that doctors usually focus on (physical objects and objective knowledge).^3^

I'm pleased that Dr Dignan is not pushing us all to be poets (I might rebel). Instead, starting from a traditional learning hierarchy of medicine–psychiatry–subspecialty–specific job,^4^ she adds another layer. She proposes that the medical humanities – a vast conglomeration of fields – is a rich hunting ground for broadening experiences. Not only rich, but necessary: I am going slightly beyond her words here, but she seems to believe that exploring the humanities makes us better people, more complete and more able to relate to others.

Dignan's aims are commendable and her poem is gorgeous. By publishing it, some of the memories she shared with her grandmother will endure for as long as this journal, perhaps in a cloud on a distant planet. Her point deserves to be applauded, explored and amplified.

This commentary started with the question of which kind of doctor serves patients best. We serve patients directly, but also indirectly by serving the systems around them. In the UK, the General Medical Council (GMC) says that a good doctor will ‘make the care of your patient your first concern.’^5^ But not your only concern. ‘Good medical practice’ also includes duties to our own knowledge and skills; to other patients; colleagues; our personal health; the medical profession; and the public. I will comment on a few of these that relate to Dignan's article.

## What's best for our skills?

I'm convinced that everyone becomes usefully dubious of their own intuitions if they regularly spend time with *Thinking, Fast and Slow*.^6^ Kahneman, and many others, have demonstrated that one of the most pervasive of all thinking defects is narrow framing, in other words failing to think broadly enough about a problem.

So for what it's worth, here are some of my favourite thought-broadening activities for psychiatrists, admittedly evidence-light. (a) We could learn from psychologists, many of whom are explicitly trained to become ‘scientist practitioners’. It's a lifelong challenge.^7,8^ (b) Some of my best colleagues love long-distance holidays. Who knows which came first? They may have been broad-minded even before the first long-haul trip. (c) In my job, watching two siblings bicker and play can produce a delightful impression of them as parts of the stream of social humanity. It also produces useful objective observations. But are these valuable enough to justify the second child missing school to attend? For the impression, no. But for the observations: perhaps for one session and not subsequently. (d) As Francis Bacon noted in 1597, ‘Reading maketh a full man, conference a ready man, and writing an exact man’. Of course, all three parts are relevant to a modern psychiatrist. Reading autobiographies and complex fiction can extend the range of people we empathise with.^9^ And, no doubt, by ‘conference’ he was referring to respectful participation in a multidisciplinary team.

## What's best for us emotionally?

Many doctors need a protective carapace, so they don't become too dispirited by demanding patients. Others need to suppress their enthusiasm for over-engaging with patients. Other doctors (one thinks particularly of researchers) need to give full flow to their meticulous tendencies. Overall, we should try to find a happy, productive, middle course that works for us personally.^10^ Part of this might include keeping a personal diary – for ourselves and those who come after us.

Dignan's aspiration is that we should all follow her example, and become more empathic. I hope some of us do. But I'm not optimistic. The classic example is the people travelling to give a lecture on good Samaritans, who step over a sick person, especially if they feel rushed.^11^ Allow me to give a personal anecdote of my own failure to transfer attitudes between relationships. During my time as a junior psychiatrist I felt sympathy towards a man whose Huntington's disease destroyed the relationships in his family: but I didn't notice the same process happening in my own family at the same time (due to my father's multiple sclerosis).

## What's best for healthcare funders?

Dignan advocates that we should spend more time with patients. In my experience, most doctors wish this. But how much time is justified? In many units, a ward assistant will be given the role of making patients feel comfortable and listened to; some of them have a delightful, naturally reassuring manner that hardly relies on personal knowledge. Usually, the history taking is delegated to junior doctors. That system creates good histories, good relationships and good training.

But the more senior doctors need to be quick, more distanced, less emotionally involved. It sounds like a shortcoming, but it isn't: their distance makes a necessary contribution to the thinking of a diverse team. The older psychiatrists typically have a wider social circle and have known vastly more patients: they have all exceeded the Dunbar number of 150 people that we can personally relate to.^12^ Even more distance is needed by doctors in poor countries: if they have only 5 or 10 minutes per patient, constant personal involvement will overwhelm them.

## What's best for the future of healthcare?

As a field, we need different things: practitioners exploring literally everywhere for insights, with the more distant explorers widely spread out, not following others, to cover the vast, less promising terrain. Even after our explorers have found a promising activity, as Dignan has done, it's fiendishly difficult to prove that it's worthwhile.^13^

We discussed these issues in our continuing professional development (CPD) group recently. One consultant said it was only her impending retirement that made her feel safe enough to say that getting to know the patients was highly undervalued, and actually disapproved of by managers. Another felt it had to be conducted as a ‘guerilla activity’ because managers saw it as detracting from the necessary work. The group found art and writing activities useful for patients, but there were no takers for creating any form of art themselves.

## Data Availability

Data availability is not applicable to this article as no new data were created or analysed in this study.

## About the author

**Jonathan Williams** is a Consultant Child and Adolescent Psychiatrist with NHS North Central London, UK.

## References

[1] Dignan CR. Evensong: how the medical humanities can strengthen a patient-centred approach to both physical and mental health conditions. BJPsych Bull YEAR; VOL: PP. 10.1192/bjb.2021.3 PMC9346493 34819197

[2] Fleming N. The tree in the quad. Am Philos Q 1985; 22: 25–36.

[3] Popper KR, Eccles JC. The Self and its Brain: An Argument for Interactionism. Springer, 1977: 36–50.

[4] Bamrah JS, Jackson R. Good Psychiatric Practice: Continuing Professional Development (College Report CR157). Royal College of Psychiatrists, 2010.

[5] Gerneral Medical Council. Good Medical Practice. GMC, 2020 (https://www.gmc-uk.org/ethical-guidance/ethical-guidance-for-doctors/good-medical-practice).

[6] Kahneman D. Thinking, Fast and Slow. Macmillan, 2011.

[7] Blair L. A critical review of the scientist-practitioner model for counselling psychology. Couns Psychol Rev 2010; 25: 19–30.

[8] Shin JH, Haynes RB, Johnston ME. Effect of problem-based, self-directed undergraduate education on life-long learning. CMAJ 1993; 148: 969–76. 8257470 PMC1490700

[9] Williams J, Hill PD. The Art of Child and Adolescent Psychiatry. Cambridge University Press, 2022.

[10] Hanoch Y, Vitouch O. When less is more: information, emotional arousal and the ecological reframing of the Yerkes-Dodson law. Theory Psychol 2004; 14: 427–52.

[11] Darley JM, Batson CD. “From Jerusalem to Jericho”: a study of situational and dispositional variables in helping behavior. J Pers Soc Psychol 1973; 27: 100–8.

[12] Hill RA, Dunbar RI. Social network size in humans. Hum Nat 2003; 14: 53–72. 26189988 10.1007/s12110-003-1016-y

[13] Spielmans GI, Pasek LF, McFall JP. What are the active ingredients in cognitive and behavioral psychotherapy for anxious and depressed children? A meta-analytic review. Clin Psychol Rev 2007; 27: 642–54. 17368886 10.1016/j.cpr.2006.06.001

